# The Effect of an eHealth Coaching Program (Smarter Pregnancy) on Attitudes and Practices Toward Periconception Lifestyle Behaviors in Women Attempting Pregnancy: Prospective Study

**DOI:** 10.2196/39321

**Published:** 2023-01-31

**Authors:** Batoul Hojeij, Sam Schoenmakers, Sten Willemsen, Lenie van Rossem, Andras Dinnyes, Melek Rousian, Regine PM Steegers-Theunissen

**Affiliations:** 1 Department of Obstetrics and Gynecology Erasmus MC University Medical Center Rotterdam Netherlands; 2 BioTalentum Ltd Godollo Hungary; 3 Department of Physiology and Animal Health Institute of Physiology and Animal Nutrition Hungarian University of Agriculture and Life Sciences Godollo Hungary

**Keywords:** diet, lifestyle, attitudes, practices, eHealth, pregnancy, Smarter Pregnancy

## Abstract

**Background:**

Lifestyle behaviors during the periconception period contribute to achievement of a successful pregnancy. Assessment of attitudes and practices toward these modifiable behaviors can aid in identifying gaps in unhealthy lifestyle behaviors with impact on intervention effectiveness.

**Objective:**

This study investigates the effectiveness of coaching by the eHealth program Smarter Pregnancy during the periconception period on improvement of attitudes and practices toward fruit and vegetable intake and smoking in women attempting pregnancy through assisted reproductive technology (ART) or natural conception.

**Methods:**

Women attempting pregnancy through ART (n=1060) or natural conception (n=631) were selected during the periconception period. The intervention groups, conceived through ART or naturally, received Smarter Pregnancy coaching for 24 weeks, whereas the control group conceived through ART and did not receive coaching. Attitudes and practices at baseline and follow-up periods were obtained from self-administered online questionnaire provided by the program. Attitudes were assessed in women with unhealthy behaviors as their intention to increase their fruit and vegetable intake and to quit smoking using a yes/no question. Outcomes on practices, suggesting effectiveness, included daily fruit (pieces) and vegetable (grams) intake, and if women smoked (yes/no). Changes in attitudes and practices were compared at 12 and 24 weeks with baseline between the ART intervention and ART control groups, and within the intervention groups between ART and natural conception. Changes in practices at 12 and 24 weeks were also compared with baseline between women with negative attitude and positive attitude within the intervention groups: ART and natural conception. Analysis was performed using linear and logistic regression models adjusted for maternal confounders and baseline attitudes and practices.

**Results:**

The ART intervention group showed higher vegetable intake and lower odds for negative attitudes toward vegetable intake after 12 weeks (β_adj_=25.72 g, *P*<.001; adjusted odds ratio [OR_adj_] 0.24, *P*<.001) and 24 weeks of coaching (β_adj_=23.84 g, *P*<.001; OR_adj_ 0.28, *P*<.001) compared with ART controls. No statistically significant effect was observed on attitudes and practices toward fruit intake (12 weeks: *P*=.16 and .08, respectively; 24 weeks: *P*=.16 and .08, respectively) and smoking behavior (12 weeks: *P*=.87; 24 weeks: *P*=.92). No difference was observed for the studied attitudes and practices between the ART intervention and natural conception intervention groups. Women with persistent negative attitude toward fruit and vegetable intake at week 12 showed lower fruit and vegetable intake at week 24 compared with women with positive attitude (β_adj_=–.49, *P*<.001; β_adj_=–30.07, *P*<.001, respectively).

**Conclusions:**

The eHealth Smarter Pregnancy program may improve vegetable intake–related attitudes and practices in women undergoing ART treatment. Women with no intention to increase fruit and vegetable intake had less improvement in their intakes. Despite small changes, this study demonstrates again that Smarter Pregnancy can be used to improve vegetable intake, which can complemented by blended care that combines face-to-face and online care to also improve fruit intake and smoking behavior.

## Introduction

The periconception period is a critical time window for the achievement of optimal reproductive and pregnancy outcomes [1-4]. Fruit and vegetable intake and smoking throughout this period can impact chance of conception, fetal growth, and neonatal outcomes [5-8]. Moreover, parents shape and direct lifestyle behaviors of their offspring that affect individual health throughout the life course [9,10].

Previous research showed that low fruit and vegetable intake, and even smoking are still common among pregnant women [11-14]. In addition, pregnant women have negative attitudes toward changing unhealthy lifestyle behaviors, which can be due to the lack of knowledge about the harms of unhealthy lifestyle behaviors, misconceptions, food taboos, and lower education level [14-16]. In accordance with the Theory of Planned Behavior, changes in fruit and vegetable intake and smoking behavior can be mediated by changes in personal attitude toward these behaviors [17-19]. Although attitudes can positively or negatively influence or predict a behavior of an individual, not all attitudes are effectively translated into practices [10,20,21]. For example, a study in Nepal showed that 45% of mothers had positive attitudes regarding their own diet and physical activity and the diet and physical activity of their children, while 90% had poor practice [10].

Changing attitudes and practices to promote a healthy diet, including adequate fruit and vegetable intake and quitting smoking, have been a potential target for public health interventions to support women attempting pregnancy in adopting healthy lifestyle behaviors [22-24]. These interventions, preferably commencing from the periconception period, were primarily based on face-to-face counseling sessions and have shown effectiveness in improving lifestyle behaviors [23,25,26]. By contrast, the use of eHealth has shown potential to be a more efficient approach and to simultaneously reduce the cost, time, and effort required from the health care professionals [27,28]. Several eHealth interventions have been implemented to promote healthy lifestyle and were effective in improving lifestyle behaviors [29,30]. For example, a short-term online intervention was effective at increasing fruit and vegetable intake in adults [29].

The web-based Smarter Pregnancy eHealth program, launched in 2011, provides information to raise awareness and empower users to adopt and maintain healthy lifestyle behaviors based on the knowledge, attitude, and practice (KAP) concept [31]. Smarter Pregnancy is designed for women as well as their (male) partners during the periconception period and aims to improve pregnancy chance and conditions as well as offspring health [31]. The effectiveness of the program has previously been established in terms of the percentage of the full 24 weeks’ program completion and improvement of lifestyle behaviors after the use of Smarter Pregnancy [32-34]. However, the program effects on attitudes toward these lifestyle behaviors have not been studied before.

In this study, we investigated the effectiveness of the eHealth coaching program Smarter Pregnancy to accomplish improvement in attitudes and practices, specifically aimed at fruit and vegetable intake and smoking after 12 and 24 weeks of coaching in women attempting pregnancy through assisted reproductive technology (ART). We also compared changes in these attitudes and practices between women attempting pregnancy through ART and natural conception. Of particular interest, we have examined the association between the attitude of women that received the Smarter Pregnancy coaching and the change in fruit and vegetable intake and smoking behavior.

## Methods

### Ethics Approval

This paper represents a retrospective analysis of a prospective study of data collected on Smarter Pregnancy subscribers that participated in a previous survey [2], a randomized controlled trial (RCT) [32], and the Rotterdam Periconception Cohort [35] between 2012 and 2019. The protocols of the 3 previous studies were approved by the Medical Ethical and Institutional Review Board at the Erasmus MC, University Medical Center, Rotterdam, the Netherlands (MEC-2011–524, MEC-NL40414.078.12, MEC-2004-227) [2,32,35]. A written, digital, or both, informed consent was obtained from all participants.

### Recruitment

**Figure 1:**
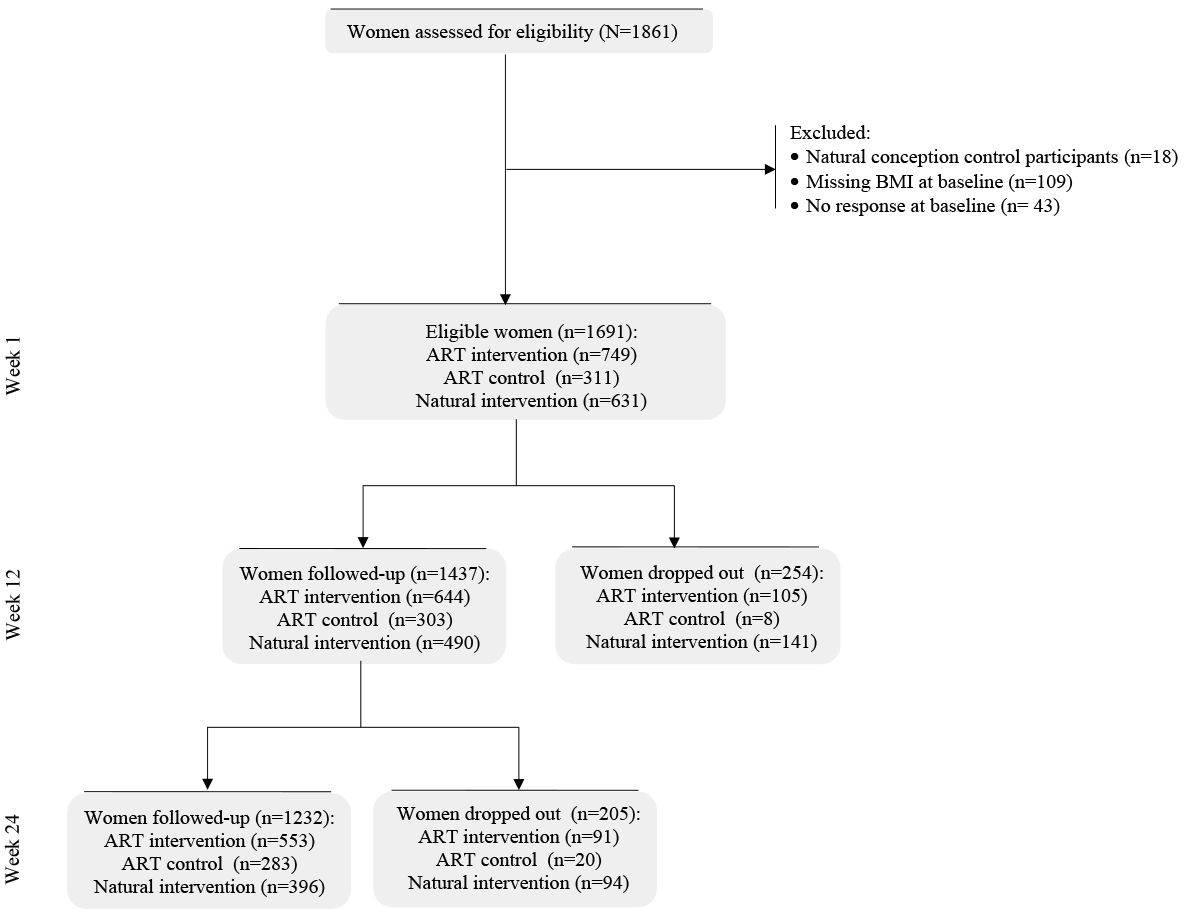
Flow chart of the study population. ART: assisted reproductive technology.

This study included 1691 women of at least 18 years of age and planning to become pregnant through ART treatment or natural conception. All participants were classified into 3 groups based on Smarter Pregnancy coaching and mode of conception: (1) the ART intervention group, which consists of women attempting to conceive via ART that received Smarter Pregnancy coaching and were participants of the survey, RCT, and cohort [2,32,35]; (2) the ART control group, which consists of women attempting to conceive via ART who did not receive Smarter Pregnancy coaching and were participants of the RCT [32]; and (3) the natural conception intervention group, which consists of women attempting to conceive naturally that received Smarter Pregnancy coaching and were participants of the survey and cohort [2,35]. Exclusion criteria of studies were pregnancy (survey), incomplete subscription and data entry (survey), oocyte donation (RCT), and following a special diet (RCT) [2,32]. In this study, women were additionally excluded if they had no response or missing BMI at baseline, or were naturally conceived controls due to small sample size (Figure 1) [33].

### Smarter Pregnancy Coaching

Detailed information on Smarter Pregnancy was described before and can also be found on the program website [31,36]. Data on baseline attitudes and practices toward fruit and vegetable intake and smoking among participants in the intervention and control groups were obtained from self-administered online questionnaire provided by the program. The questionnaire also assessed the women’s weight, height, and if they were pregnant. The ART and natural conception intervention groups were coached for 24 weeks on fruit and vegetable intake and smoking via weekly emails and push emails with feedback, recommendations, tips, vouchers, and seasonal recipes to educate and encourage adopting healthy behaviors based on the identified inadequate behaviors at baseline and tailored for women who become pregnant. Data on changes in attitudes and practices toward these lifestyle behaviors of the ART and natural conception intervention groups were obtained at 6, 12, 18, and 24 weeks of follow-up using the same baseline questionnaire. The ART control group did not receive any coaching nor feedback on their lifestyle behaviors, apart from 1 seasonal recipe per week to maintain adherence to program enrollment to be followed up at 12 and 24 weeks using the same baseline questionnaire.

### Variables and Outcomes

All women were requested to fill in their practices at each follow-up period. Being designed for implementation also in a clinical setting, dietary intake of fruits and vegetables was assessed using a closed-ended, brief dietary questionnaire as part of the self-administered online questionnaire. Smoking behavior was assessed using a yes/no question. Women were asked to report their daily intake of fruits (in number of juice glasses and pieces of fruit) and vegetables (in number of serving spoons of raw and cooked vegetables) over the past week with a 7-point scale for duration (0 to 7 days) and a 6-point scale for frequency (1 to ≥5 servings). Adequate fruit intake per day was defined as ≥2 pieces/day and adequate vegetable intake per day was defined as ≥200 g/day, calculated according to the following formulas:



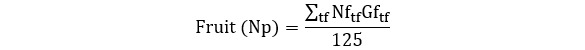





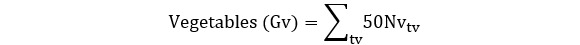



where Np is the number of standard fruit pieces; tf represents the type of fruit (large, mandarin, other fruits, and juice); Nf is the number of juice glasses or pieces of fruit; Gf is the grams of fruit in each fruit type; Gv is the grams of vegetables; tv represents the type of vegetable (raw and cooked); and Nv is the number of serving spoons of raw or cooked vegetables. A standard fruit piece was estimated as 125 g of fruit and a serving spoon of raw or cooked vegetables was estimated as 50 g of vegetables.

To assess attitudes, women were inquired for their readiness to adopt a positive behavior based on a yes/no question for fruit (ie, “Are you planning to eat at least 2 pieces of fruits every day?”) and vegetable intake (ie, “Are you planning to eat at least 200 g of vegetables every day ?”), and for smoking (ie, “Would you stop?”). Attitude questions were only asked for women with identified inadequate behavior at baseline and during the follow-up periods. Thus, women with inadequate fruit (<2 pieces/day) and vegetable (<200 g/day) intake and smokers at baseline were considered for assessment of program effectiveness on attitudes. Women that answered “yes” to the attitude question or with adequate behavior during follow-up periods were categorized as “positive,” whereas those that answered “no” were categorized as “negative.”

### BMI Assessment

BMI (kg/m^2^) was calculated based on self-reported weight (kg) and height (cm) from the Smarter Pregnancy program and women were classified as obese (BMI ≥30.0 kg/m^2^), overweight (BMI 25.0-29.9 kg/m^2^), normal weight (BMI 18.5-24.9 kg/m^2^), or underweight (<18.5 kg/m^2^) [37].

### Statistical Analysis

Baseline maternal characteristics were compared between the ART intervention and ART control groups and between the ART intervention and natural conception intervention groups using chi-square test for categorical variables and Mann-Whitney *U* test for continuous variables. Program completion was calculated based on the number of women that completed the 24 weeks’ follow-up questionnaire. All women were included in the analysis of dietary and smoking-related attitudes and practices regardless of dropout or completion of the program.

To assess whether Smarter Pregnancy coaching of subfertile women attempting pregnancy is effective in improving their attitudes and practices toward fruit and vegetable intake and smoking during the periconception period, changes were compared between the ART intervention and ART control groups at the mid (12 weeks) and end of coaching periods (24 weeks). We have also compared the changes in these attitudes and practices between the ART intervention and natural conception intervention groups at 12 and 24 weeks. Linear regression models adjusted for age, BMI, pregnancy (yes/no), and baseline intake were used to compare changes in fruit and vegetable intake. Change in smoking behavior was compared using logistic regression adjusted for age, BMI, and pregnancy (yes/no). To compare changes in attitudes toward fruit and vegetable intake and smoking cessation, logistic regression models adjusted for age, BMI, pregnancy (yes/no), and baseline attitudes were used.

To analyze the association between attitudes and the change in practice after 12 and 24 weeks of coaching, changes in fruit and vegetable intake as well as smoking were compared between women with negative attitude and women with positive attitude in both the ART intervention group and the natural conception intervention group. For fruit and vegetable intake, linear regression models adjusted for age, BMI, pregnancy (yes/no), baseline intake, and conception mode (ART/natural) were used. Smoking was analyzed using the logistic regression model adjusted for age, BMI, pregnancy (yes/no), and conception mode (ART/natural).

Missing data of the outcome variables and covariates at baseline (range <1%-9%), 12 weeks (range <1%-31%), and 24 weeks (range <1%-38%) were handled using multiple imputations of 5 generated imputation sets for 10 iterations each. Continuous variables were imputed using predictive mean matching and categorical variables were imputed using (polytomous) logistic regression.

Multiple imputations were performed using R (version 4.1.1 for Windows). All analyses were performed using SPSS (version 21.0 for Windows; IBM Corp.) and *P* values <.05 were considered statistically significant.

## Results

### Characteristics of Study Population

The baseline characteristics of each study group are depicted in Table 1. The intervention group consisted of 749 women conceiving through ART and 631 naturally, while 311 women were included in the ART control group. Of the total population (N=1691), 1232 (72.86%) women completed the program and 549 (32.47%) women participated with their male partner. The median age of women was 32 years and the median BMI was in the normal range (24 kg/m^2^).

In the overall population (N=1691), 51.51% (n=871) and 76.94% (n=1301) of women showed, respectively, inadequate fruit (<2 pieces/day) and vegetable intake (<200 g/day), and 11.06% (n=187) smoked. Negative attitudes were reported in 22.27% (194/871) and 19.98% (260/1301) of these women, respectively, toward increasing fruit and vegetable intake, and in 10.2% (19/187) for smoking cessation. Besides, the ART intervention group showed less intention to improve vegetable intake (*P*=.02) at baseline, which represents the onset of Smarter Pregnancy activation, compared with the natural conception intervention group.

**Table 1:** Baseline characteristics of the study participants of the ART^a^ intervention, ART control, and natural conception intervention groups.

Maternal characteristics			ART intervention (n=749)		Missing, n (%)		ART control (n=311)		Missing, n (%)		*P* value^b^		Natural conception intervention (n=631)		Missing, n (%)		*P* value^c^
Age (years), median (IQR)			32.76 (29.14-36.62)		12 (1.6)		33.27 (30.43-36.84)		3 (1.0)		.04		30.23 (27.35-33.81)		6 (1.0)		<.001
BMI (kg/m^2^), median (IQR)			23.94 (5.90)				23.78 (4.77)				.39		23.34 (5.67)				.06
**BMI categories, n (%)**																	
	Obese	102 (13.6)				29 (9.3)				.23		90 (14.3)				.25	
	Overweight	182 (24.3)				80 (25.7)						179 (28.4)					
	Normal weight	448 (59.8)				192 (61.7)						345 (54.7)					
	Underweight	17 (2.3)				10 (3.2)						17 (2.7)					
	Pregnant, n (%)	0 (0)				0 (0)						0					
	Partner participation, n (%)	276 (36.8)				112 (36.0)				.80		161 (25.5)				<.001	
	Program completion, n (%)	553 (73.8)				283 (91.0)				<.001		396 (62.8)				<.001	
**Practices, n (%)**																	
	Inadequate vegetable intake	570 (76.1)		1 (0.1)		223 (71.7)				.12		508 (80.5)				.05	
	Inadequate fruit intake	363 (48.4)		5 (0.7)		169 (54.3)		4 (1.3)		.07		339 (53.7)		1 (0.2)		.06	
	Smokers	96 (12.8)		7 (0.9)		20 (6.4)		6 (1.9)		.003		71 (11.3)		1 (0.2)		.35	
**Negative attitudes, n (%)**																	
	Vegetable intake–related attitude^d^	125 (21.9)				53 (23.8)				.58		82 (16.1)				.02	
	Fruit intake–related attitude^e^	67 (18.4)				44 (26.0)				.045		83 (24.5)				.05	
	Smoking attitude^f^	9 (9.6)				0 (0)				.16		10 (14.5)				.33	

^a^ART: assisted reproductive technology.

^b^ART intervention versus ART control.

^c^ART intervention versus natural conception intervention.

^d^For the ART intervention, ART control, and natural conception intervention n=570, 223, and 508, respectively.

^e^For the ART intervention, ART control, and natural conception intervention n=363, 169, and 339, respectively.

^f^For the ART intervention, ART control, and natural conception intervention n=94, 19, and 69, respectively.

### Impact of Smarter Pregnancy on Modifying Negative Attitudes and Unhealthy Practices

The ART intervention group showed greater improvement in vegetable intake at 12 (β_adj_=25.72 g, *P*<.001) and 24 weeks (β_adj_=23.84 g, *P*<.001) compared with the ART control group (Table 2). Moreover, compared with the ART control group, the ART intervention group showed greater reduction in negative attitudes toward increasing vegetable intake at 12 (adjusted odds ratio [OR_adj_] 0.24, *P*<.001) and 24 weeks (OR_adj_ 0.28, *P*<.001; Table 2). In the adjusted model, no effect of Smarter Pregnancy coaching was observed for improving attitudes and practices toward fruit intake at 12 (*P*=.08 and .16, respectively) and 24 (*P*=.08 and .16, respectively) weeks (Table 2). Furthermore, no effect was observed on improving smoking behavior (12 weeks: *P*=.87; 24 weeks: *P*=.92; Table 2).

No significant differences were observed between the ART and natural conception intervention groups at 12 and 24 weeks in any of the studied attitudes and practices toward fruit (12 weeks: *P*=.71 and .39, respectively; 24 weeks: *P*=.60 and .10, respectively) and vegetable (12 weeks: *P*=.80 and .33, respectively; 24 weeks: *P*=.67 and .75, respectively) intake and smoking (12 weeks: *P*=.59 and .87, respectively; 24 weeks: *P*=.44 and .86, respectively) (adjusted model; Multimedia Appendix 1).

**Table 2:** Change in attitudes and practices toward dietary intake and smoking^a^ in the ART^b^ intervention group compared with the ART control group after 12 and 24 weeks of Smarter Pregnancy program enrollment.

Maternal lifestyle behaviors				Crude				Adjusted^c^		
				Week 12		Week 24		Week 12		Week 24
**Practices**										
	**Vegetable^d^ (g)**									
		β^e^ (95% CI)	21.16 (9.50 to 32.81)		19.85 (9.02 to 30.69)		25.72 (16.56 to 34.87)		23.84 (15.07 to 32.60)	
		*P* value	<.001		<.001		<.001		<.001	
	**Fruit^d^ (pieces)**									
		β (95% CI)	0.18 (–0.06 to 0.43)		0.23 (–0.00 to 0.46)		0.13 (–0.05 to 0.32)		0.14 (–0.05 to 0.33)	
		*P* value	.14		.05		.16		.16	
	**Smoking^f,g^**									
		OR^h^ (95% CI)	0.87 (0.31 to 2.39)		0.94 (0.36 to 2.50)		0.91 (0.31 to 2.71)		0.95 (0.32 to 2.82)	
		*P* value	.78		.91		.87		.92	
**Negative attitudes**										
	**Vegetable^i^**									
		OR^j^ (95% CI)	0.40 (0.26 to 0.60)		0.42 (0.28 to 0.64)		0.24 (0.14 to 0.43)		0.28 (0.16 to 0.49)	
		*P* value	<.001		<.001		<.001		<.001	
	**Fruit^k^**									
		OR (95% CI)	0.47 (0.27 to 0.81)		0.45 (0.24 to 0.82)		0.56 (0.29 to 1.08)		0.51 (0.24 to 1.09)	
		*P* value	.006		.009		.08		.08	

^a^Model was not able to estimate the effect of Smarter Pregnancy coaching on negative attitude toward smoking cessation due to the absence of women with negative attitude in the ART control group.

^b^ART: assisted reproductive technology.

^c^Model adjusted for age, BMI, pregnancy, and corresponding baseline attitudes and practices.

^d^For the ART intervention and ART control groups n=749 and 311, respectively.

^e^β indicates the difference in change of practice in the ART intervention group compared with the ART control group.

^f^For the ART intervention and ART control groups n=96 and 20, respectively.

^g^Baseline smoking behavior is not included as covariate in the adjusted model.

^h^Odds ratio for smoking.

^i^For the ART intervention and ART control groups n=570 and 223, respectively.

^j^Odds ratio for negative attitude in women with inadequate behavior of the ART intervention group compared with the ART control group.

^k^For the ART intervention and ART control groups n=363 and 169, respectively.

Subgroup analysis revealed no significant differences (*P*>.05 in all cases) in the change of attitudes and practices toward fruit and vegetable intake and smoking between women with overweight/obesity compared with those with normal weight of the ART and natural conception intervention groups (adjusted model; Multimedia Appendices 2 and 3). After 24 weeks of coaching, women in the natural conception intervention group participating with their male partner showed greater improvement in vegetable intake (β_adj_=18.86 g, *P*=.02) compared with women participating alone, whereas no difference in the change was observed in the other attitudes and practices (adjusted model; Multimedia Appendices 4 and 5).

### Impact of Attitudes on Change in Practices

In the total intervention population (both ART and natural conception intervention groups), women with negative attitudes toward fruit and vegetable intake at baseline showed lower fruit (β_adj_=–.27 pieces, *P*<.001) and vegetable (β_adj_=–26.41 g, *P*<.001) intake compared with women with positive attitudes. Following coaching, women with negative attitude at baseline showed less improvement in vegetable intake at week 12 (β_adj_=–11.86 g, *P*=.02) compared with those with positive attitude, whereas no significant difference was observed between the 2 groups toward fruit intake (*P*=.09; Table 3). Furthermore, women identified with persistent negative attitude until week 12 showed less improvement in fruit (β_adj_=–.49 pieces, *P*<.001) and vegetable intake (β_adj_=–30.07 g, *P*<.001) at week 24 compared with those with positive attitude (Table 4).

In the subgroup analysis for the mode of conception, women in the ART intervention and natural conception intervention groups with negative attitudes toward increasing fruit and vegetable intake at baseline also showed lower fruit (β_adj_=–.27 pieces, *P*<.001; β_adj_=–.28 pieces, *P*<.001, respectively) and vegetable intake (β_adj_=–30.00 g, *P*<.001; β_adj_=–21.33 g, *P*<.001, respectively) compared with women with positive attitudes. Following coaching, only women in the ART intervention group with negative attitude toward increasing fruit intake at baseline showed less improvement in fruit intake at week 12 (β_adj_=–.37 pieces, *P*=.03) compared with those with positive attitude (Table 3). Furthermore, women of the ART intervention and natural conception intervention groups with persistent negative attitude until week 12 showed less improvement in fruit (β_adj_=–.57 pieces, *P*=.01; β_adj_=–.44 pieces, *P*=.02, respectively) and vegetable intake (β_adj_=–37.81 g, *P*<.001; β_adj_=–20.90 g, *P*=.02, respectively) at week 24 compared with those with positive attitude (Table 4).

Change in smoking behavior was compared at week 12 between smokers with negative attitude and smokers with positive attitude identified at baseline and showed no significant difference in the total population (*P*=.71) and in subgroup analysis for conception mode (ART: *P*=.56; natural conception: *P*=.97; Table 3).

**Table 3:** Change in dietary and smoking-related practices after 12 weeks of coaching between women with negative attitude and women with positive^a^ attitude identified at baseline of the intervention group (ART^b^ intervention and natural conception intervention).

Maternal lifestyle behaviors			Crude						Adjusted				
			ART intervention		Natural conception intervention		Total		ART intervention^c^		Natural conception intervention^c^		Total^d^
**Vegetable^e^ (g)**													
	β^f^ (95% CI)	–28.38 (–45.72 to –13.04)		–29.74 (–47.21 to –12.27)		–28.30 (–39.13 to –17.47)		–9.48 (–25.83 to 6.87)		–15.60 (–32.14 to 0.94)		–11.86 (–22.06 to –1.66)	
	*P* value	<.001		.001		<.001		.25		.06		.02	
**Fruit^g^ (pieces)**													
	β^e^ (95% CI)	–.55 (–0.90 to –0.21)		–.24 (–0.58 to 0.10)		–.40 (–0.63 to –0.16)		–.37 (–0.71 to –0.04)		–.03 (–0.36 to 0.29)		–.19 (–0.41 to 0.03)	
	*P* value	.002		.17		.001		.03		.84		.09	
**Smoker^h,i^**													
	OR^j^ (95% CI)	1.27 (0.30 to 5.46)		0.90 (0.23 to 3.56)		1.06 (0.39 to 2.86)		1.62 (0.32 to 8.13)		0.97 (0.22 to 4.35)		1.22 (0.43 to 3.43)	
	*P* value	.75		.87		.91		.56		.97		.71	

^a^Positive attitude is perceived as intention to improve a certain behavior.

^b^ART: assisted reproductive technology.

^c^Model adjusted for age, BMI, pregnancy, and baseline practice.

^d^Model adjusted for age, BMI, pregnancy, baseline practice, and conception mode.

^e^For the ART intervention, natural conception intervention, and total population n=570, 508, and 1078, respectively.

^f^β indicates the difference in change in dietary-related practices at 12 weeks between women with negative and positive attitudes.

^g^For the ART intervention, natural conception intervention, and total population n=363, 339, and 702, respectively.

^h^For the ART intervention, natural conception intervention, and total population n=96, 71, and 167, respectively.

^i^Baseline practice was not included in the adjusted model.

^j^Odds ratio for smoking.

**Table 4:** Change in dietary intake after 24 weeks of coaching between women with persistent negative attitude and women with positive^a^ attitude identified at week 12 of the intervention group (ART^b^ intervention and natural conception intervention).^c^

Maternal lifestyle behaviors			Crude							Adjusted						
			ART intervention		Natural conception intervention		Total		ART intervention^d^			Natural conception intervention^d^		Total^e^		
**Vegetable^f^ (g)**																
	β^g^ (95% CI)	–45.41 (–62.03 to –28.78)		–32.84 (–50.85 to –14.83)		–39.64 (–51.41 to –27.86)		–37.81 (–54.21 to –21.42)			–20.90 (–38.31 to –3.48)		–30.07 (–41.32 to –18.81)			
	*P* value	<.001		<.001		<.001		<.001			.02		<.001			
**Fruit^h^ (pieces)**																
	β (95% CI)	–.74 (–1.18 to –0.31)		–.63 (–1.07 to –0.19)		–.69 (–1.04 to –0.33)		–.57 (–0.99 to –0.14)			–.44 (–0.80 to –0.09)		–.49 (–0.79 to –0.20)			
	*P* value	.001		.007		.001		.01			.02		.002			

^a^Positive attitude is perceived as intention to improve a certain behavior.

^b^ART: assisted reproductive technology.

^c^The model was not able to estimate outcomes on smoking behavior due to the absence of women with negative attitude in smokers.

^d^Model adjusted for age, BMI, pregnancy, and baseline practice.

^e^Model adjusted for age, BMI, pregnancy, baseline practice, and conception mode.

^f^For the ART intervention, natural conception intervention, and total population n=399, 376, and 775, respectively.

^g^β indicates the difference in change of dietary-related practices at 24 weeks between women with negative and positive attitudes.

^h^For the ART intervention, natural conception intervention, and total population n=191, 204, and 395, respectively.

## Discussion

### Principal Findings

In this study, we have shown that negative attitudes and unhealthy practices toward lifestyle behaviors are present among subfertile women with an indication for ART and fertile women attempting pregnancy. Findings of our study show that the majority of women had inadequate fruit intake (871/1691, 51.51%) and vegetable (1301/1691, 76.94%) intake, and 11.06% (187/1691) smoked. Absence of intention to improve fruit and vegetable intake was present in 22.27% (194/871) and 19.98% (260/1301) of these women, respectively, and in 10.16% (19/187) for smoking cessation. The use of eHealth coaching using Smarter Pregnancy was associated with improvement in vegetable intake–related attitudes and practices among women planning to undergo ART treatment. Besides, we found no difference in changes in the studied attitudes and practices between the ART and natural conception groups that received Smarter Pregnancy coaching. Interestingly, women with persistent negative attitude toward increasing fruit and vegetable intake that received Smarter Pregnancy coaching showed less improvement in their intake compared with women with positive attitude.

### Outcomes Interpretation and Comparison With Other Studies

We have found that more than half of the population had inadequate fruit and vegetable intake. Results are consistent with a study by Landais et al [38], which was performed in Moroccan women of childbearing age that showed comparable proportions of women with less-than-recommended intake of fruits and vegetables [38]. eHealth-based coaching using Smarter Pregnancy was associated with improvement in vegetable intake–related attitudes and practices, throughout and at the end of the program. Outcomes were comparable to a previous RCT that showed an effect of Smarter Pregnancy on improving vegetable intake [33]. We found no effect of Smarter Pregnancy on improving fruit intake as well as fruit intake–related attitude, which emphasizes on the relationship between attitudes and practices. The coaching on vegetable intake is more intensive as compared with that on fruit intake, which can in part explain the absence of effect of the program on improving fruit intake. Determinants of fruit and vegetable intake are cost, economic status, education level, knowledge, social norms and support, and household environment [38-41]. Motivating women attempting pregnancy to increase their fruit and vegetable intake is recommended as it is associated with favorable pregnancy conditions, such as reduced risk of preeclampsia, gestational diabetes mellitus, and upper respiratory tract infection [42-44]. Furthermore, increased fruit intake can reduce the risk of endometriosis and time to pregnancy, which can be a potential concern in the subfertile population [5,45,46]. Furthermore, higher vegetable intake can increase the probability of clinical pregnancy and reduce the risk of having small-for-gestational-age newborn [5,47].

Findings on smoking behavior showed that 11.06% (187/1691) of women smoked, which is comparable to 2 other studies addressing smoking behavior in women preconceptionally [48]. Empowering women attempting pregnancy to quit smoking, particularly the ART population, is recommended as smoking is associated with a lower in vitro fertilization success rate, pregnancy chance, and live birth rate as well as increased risk for spontaneous abortion [49,50]. Women’s awareness about the effect of smoking on fertility apart from Smarter Pregnancy coaching [51], the low number of smokers (n=20), and the absence of negative attitude toward smoking cessation in the control group might have affected outcomes of the effectiveness of Smarter Pregnancy on smoking cessation. By contrast, a previous RCT including 111 nonsmokers and 10 smokers showed that Smarter Pregnancy lowered the risk of smoking in the subgroup of women with overweight/obesity [52].

Inadequate dietary behaviors can be in part explained by the perceived negative attitudes as they were associated with less intake at the start of the program in the total intervention group of fertile and subfertile women. Results were consistent with 2 other studies that showed a relationship between dietary-related attitudes and practices [40,53]. Based on the Theory of Planned Behavior, practices of certain behavior can be predicted by attitudes [17]. In this study we have demonstrated this theory, as we have seen that women with no intention to increase their fruit and vegetable intake had less improvement in their intake across follow-up periods. However, that was not observed with smoking behavior, as we did not observe an association between intention to quit smoking and smoking cessation. Tobacco addiction due to its nicotine content and withdrawal symptoms can make smoking cessation a hard practice to achieve, even if intentions to quit smoking are present in smokers [54]. Besides, socially desirable answers and the low number of women with negative attitudes (n=19) might have influenced the outcomes on the relationship between attitude and practice toward smoking cessation.

Women conceiving via ART hypothetically have a higher intention to change their lifestyle behaviors to improve their pregnancy chance [55]. However, in this study, we have found no differences in attitudes and practices toward fruit and vegetable intake and smoking between women attempting pregnancy through ART and naturally. It might be that women of the ART group did not observe these factors as major contributors to pregnancy chance, for example, due to unawareness, particularly toward the contribution of smoking to infertility [56,57]. However, assessment of women awareness was not an outcome of this study. In the subgroup analysis for BMI, we did not find a difference in change in attitudes and practices after coaching between women with overweight/obesity and women with normal BMI. Our outcomes are comparable to an RCT that showed no difference in Smarter Pregnancy effect on fruit and vegetable intake between BMI groups [52]. Information on the effectiveness of behavioral lifestyle interventions between different BMI categories and conception modes is scarce and requires further investigation for a targeted management of these groups.

### Implications for Clinical Practice

The study has shown that unhealthy practices and negative attitudes are present among both subfertile women who underwent ART and fertile women attempting pregnancy that can affect fertility status and pregnancy outcomes, indicating the importance of improving daily lifestyle-related attitudes and practices in this population. This can be achieved through commercials, billboards, public policies, and implementing interventions targeting lifestyle behaviors such as blended care. For example, a well-implemented eHealth coaching program can be used as an alternative tool for face-to-face counseling as it saves effort and time for the participant as well as for the counselor [27].

The concept of clinical significance is not well defined and can be subjective depending on individual and goals [58]. In this study we considered a 15-32-g increase in daily vegetable intake to be a meaningful change, particularly among women with inadequate intake (570/749, 76.1%, in the ART intervention group), as they will start to approach the recommended daily intake. In terms of health outcomes, previous studies showed that small increases in vegetable intake (eg, 10-20 g/day) were associated with increased newborn head circumference and birth length, as well as reduced risk of diabetes type II [59,60]. Moreover, according to Rose’s prevention paradox, “a preventive measure that brings large benefits to the community offers little to each participating individual” [61]. Therefore, our results suggest that the eHealth Smarter Pregnancy coaching program can be used to improve dietary vegetable intake, particularly through improving negative attitudes in women undergoing ART treatment. Implementation of Smarter Pregnancy to improve vegetable intake is recommended and can be complemented with blended care to also help improve fruit intake and smoking cessation. Besides, improvement in vegetable intake–related attitude suggests that the improvement in vegetable intake can be maintained for a longer period, even after the 24-week coaching period. By contrast, women with negative attitude might require more coaching to improve their dietary intake; therefore, a tailored coaching program according to user attitude is recommended in interventions targeting dietary behaviors. Furthermore, identifying factors hindering users from achieving healthy behaviors, for example, by including questions assessing knowledge and socioeconomic factors, can aid in identifying areas for improvement in future program updates.

### Strengths and Limitations

To the best of our knowledge, this is one of the few studies that investigated attitudes and practices toward lifestyle behaviors, with an emphasis on eHealth coaching, in women attempting pregnancy. The effect of the intervention on attitudes and practices was studied longitudinally to distinguish changes across different follow-up periods throughout the coaching period. Another strength is that we aimed to investigate whether attitudes can affect changes in practices upon intervention, as this can help in understanding components affecting health behavior change. Besides, we have considered important sociodemographic aspects of population such as age, BMI, and pregnancy that can influence attitudes and practices.

This study has some limitations. First, absence of a control group for naturally conceiving women hindered us from assessing intervention effectiveness in this population. Second, fruit and vegetable intakes were reported using a self-administered brief dietary assessment tool by means of specified portion size and frequency using an ordinal scale, and were assessed for a short term (ie, 7 days). Therefore, dietary misreporting due to recall bias, the desire to appear compliant in the intervention groups, measurements errors, and low within-person variation are to be considered [62]. Although we did not use objective measurements, such as assessment of biomarker levels to validate dietary intake in this study, in our previously published RCT we measured serum folate levels as a concentration biomarker to validate the Smarter Pregnancy program for vegetable and fruit intake and the use of folic acid supplements [32]. The RCT has reported that higher levels of serum folate were observed in the intervention group that showed improvements in dietary risk score [32]. However, significant effects of fruit and vegetable intake separately on serum folate levels were not investigated due to limited power [32]. Moreover, we included a control group that reduces information bias, as the measurement error would be very similar in the intervention and control groups, which supports the validity of the estimates. In addition, socially acceptable answers are to be considered for smoking and the risk of recall bias for self-reported BMI. Finally, questions on attitudes and practices were extracted from the Smarter Pregnancy questionnaire and not from a KAP model, which is recommended in future studies assessing the intervention effectiveness.

### Conclusions

This study addressed attitudes and practices toward lifestyle behaviors in women attempting pregnancy through ART and naturally, with a focus on the role of the eHealth Smarter Pregnancy coaching program. Smarter Pregnancy use was associated with improvement in vegetable intake–related attitudes and practices. Moreover, negative attitudes toward improving fruit and vegetable intake were associated with less improvement in intake. Inadequate fruit and vegetable intake, and to a lesser extent, smoking were prevalent in women attempting pregnancy. Negative attitudes toward increasing fruit and vegetable intake and smoking cessation were also present in women with unhealthy behaviors. Despite small changes, our results demonstrate again that Smarter Pregnancy can be used to improve vegetable intake which can be complemented with the use of blended care that combines face-to-face and online care to also improve fruit intake and smoking cessation.

## Appendix

Multimedia Appendix 1 Difference in change in attitudes and practices towards fruit and vegetable intake and smoking between ART intervention and natural conception intervention groups after 12 and 24 weeks of Smarter Pregnancy enrollment.

Multimedia Appendix 2 Difference in change of attitudes towards fruit and vegetable intake and smoking between overweight/obese women compared to normal weight women in the ART intervention, ART control and natural conception intervention groups after 12 and 24 weeks of Smarter Pregnancy enrollment.

Multimedia Appendix 3 Difference in change of fruit and vegetable intake and smoking between overweight/obese women compared to normal weight women in the ART intervention, ART control and natural conception intervention groups after 12 and 24 weeks of Smarter Pregnancy enrollment.

Multimedia Appendix 4 Difference in change of attitudes towards fruit and vegetable intake and smoking between women participating with male partner compared to women participating alone in the ART intervention, ART control and natural conception intervention groups after 12 and 24 weeks of Smarter Pregnancy enrollment.

Multimedia Appendix 5 Difference in change in fruit and vegetable intake and smoking between women participating with male partner compared to women participating alone in the ART intervention, ART control and natural conception intervention groups after 12 and 24 weeks of Smarter Pregnancy enrollment.

## Abbreviations

ART: assisted reproductive technology
KAP: knowledge, attitude, and practice
OR: odds ratio
ORadj: adjusted odds ratio
RCT: randomized controlled trial
