# Supplementary material for: The Effect of an eHealth Coaching Program (Smarter Pregnancy) on Attitudes and Practices Toward Periconception Lifestyle Behaviors in Women Attempting Pregnancy: Prospective Study
Source: J Med Internet Res. 2023 Jan 31;25:e39321. doi: 10.2196/39321 (PMC9929732; doi:10.2196/39321)
Supplement: Multimedia Appendix 1 [file jmir_v25i1e39321_app1.docx]

## **Appendix 1**

Table S1. Difference in change in attitudes and practices towards fruit and vegetable intake and smoking between ART intervention and natural conception intervention groups after 12 and 24 weeks of Smarter Pregnancy enrollment.

|  | Crude | | Adjusted^a^ | |
| --- | --- | --- | --- | --- |
|  | Week 12 | Week 24 | Week 12 | Week 24 |
| **Practices** |  |  |  |  |
| Vegetable^b^ (grams) |  |  |  |  |
| β^c^ | 7.280 | 5.24 | 1.884 | 0.625 |
| 95% CI^d^ | 2.72, 11.84 | 0.92, 9.57 | -1.92, 5.69 | -3.21, 4.46 |
| *P* value | 0.002 | 0.02 | 0.33 | 0.75 |
| Fruit^b^ (pieces) |  |  |  |  |
| β | 0.11 | 0.13 | 0.04 | 0.08 |
| 95% CI | 0.10, 0.21 | 0.03, 0.23 | -0.05, 0.12 | -0.02, 0.19 |
| *P* value | 0.03 | 0.01 | 0.39 | 0.10 |
| Smoking^e,f^ |  |  |  |  |
| OR^g^ | 0.98 | 1.07 | 0.94 | 1.06 |
| 95% CI | 0.49, 1.95 | 0.57, 1.02 | 0.46, 1.93 | 0.54, 2.08 |
| *P* value | 0.94 | 0.83 | 0.87 | 0.86 |
| **Negative attitudes** |  |  |  |  |
| Vegetable^h^ |  |  |  |  |
| OR^i^ | 1.27 | 1.22 | 0.93 | 0.90 |
| 95% CI | 0.82, 1.96 | 0.82, 1.82 | 0.55, 1.59 | 0.56, 1.45 |
| *P* value | 0.29 | 0.33 | 0.80 | 0.67 |
| Fruit^j^ |  |  |  |  |
| OR | 0.83 | 0.73 | 1.12 | 0.84 |
| 95% CI | 0.51, 1.38 | 0.42, 1.29 | 0.62, 2.01 | 0.42, 1.65 |
| *P* value | 0.48 | 0.28 | 0.71 | 0.60 |
| Smoking^k^ |  |  |  |  |
| OR | 0.56 | 0.56 | 0.61 | 0.49 |
| 95% CI | 0.17, 1.97 | 0.16, 1.90 | 0.10, 3.64 | 0.08, 3.01 |
| *P* value | 0.38 | 0.35 | 0.59 | 0.44 |

^a^ Model adjusted for age, BMI, pregnancy and respective baseline attitudes or practices.

^b^ N of ART intervention and natural conception intervention groups, respectively: N= 749 and 631.

^c^ β: Beta coefficients for difference in change of intake.

^d^ CI: confidence interval

^e^ N of ART intervention and natural conception intervention groups, respectively: N= 96 and 71.

^f^ Baseline smoking behavior is not included as covariate in adjusted model.

^g^ OR: odds ratio for smoking.

^h^ N of ART intervention and natural conception intervention groups, respectively: N= 570 and 508.

^i^ OR: odds ratio for negative attitude.

^j^ N of ART intervention and natural conception intervention groups, respectively: N= 363 and 339.

^k^ N of ART intervention and natural conception intervention groups, respectively: N= 96 and 71.
