# Supplementary material for: The Effect of an eHealth Coaching Program (Smarter Pregnancy) on Attitudes and Practices Toward Periconception Lifestyle Behaviors in Women Attempting Pregnancy: Prospective Study
Source: J Med Internet Res. 2023 Jan 31;25:e39321. doi: 10.2196/39321 (PMC9929732; doi:10.2196/39321)
Supplement: Multimedia Appendix 2 [file jmir_v25i1e39321_app2.docx]

## **Appendix 2**

|  | Crude | | | | | | Adjusted^a^ | | | | | |
| --- | --- | --- | --- | --- | --- | --- | --- | --- | --- | --- | --- | --- |
|  |  | Week 12 | | Week 24 | | | Week 12 | | | Week 24 | | |
|  | ART INT^b^ | ART control | Natural INT | ART INT | ART control | Natural INT | ART INT | ART control | Natural INT | ART INT | ART control | Natural INT |
|  |  |  |  |  |  |  |  |  |  |  |  |  |
| **Vegetables**^c^ |  |  |  |  |  |  |  |  |  |  |  |  |
| OR^d^ | 0.39 | 1.17 | 0.88 | 0.51 | 1.13 | 0.94 | 0.50 | 0.80 | 0.98 | 0.71 | 1.03 | 1.06 |
| 95% CI^e^ | 0.19, 0.78 | 0.62, 2.217 | 0.45, 1.72 | 0.27, 0.95 | 0.60, 2.14 | 0.48, 1.81 | 0.21, 1.15 | 0.30, 2.15 | 0.40, 2.40 | 0.34, 1.51 | 0.43, 2.459 | 0.46, 2.45 |
| *P* value | 0.009 | 0.63 | 0.70 | 0.04 | 0.71 | 0.84 | 0.10 | 0.66 | 0.96 | 0.38 | 0.95 | 0.89 |
| **Fruit**^f^ |  |  |  |  |  |  |  |  |  |  |  |  |
| OR | 0.77 | 0.76 | 1.29 | 0.60 | 0.509 | 1.21 | 0.85 | 0.53 | 1.51 | 0.65 | 0.25 | 1.32 |
| 95% CI | 0.34, 1.74 | 0.33, 1.75 | 0.61, 2.69 | 0.24, 1.50 | 0.204, 1.271 | 0.55, 2.70 | 0.32, 2.21 | 0.19, 1.46 | 0.65, 3.52 | 0.23, 1.83 | 0.08, 0.84 | 0.55, 3.13 |
| *P* value | 0.53 | 0.52 | 0.51 | 0.27 | 0.51 | 0.63 | 0.73 | 0.22 | 0.34 | 0.41 | 0.02 | 0.53 |
| **Smoking**^g^ |  |  |  |  |  |  |  |  |  |  |  |  |
| OR | 0.417 | *NA*^h^ | 1.52 | 0.42 | *NA* | 1.60 | 0.27 | *NA* | 0.66 | 0.48 | *NA* | 0.62 |
| 95% CI | 0.045, 3.888 | *NA* | 0.26, 9.03 | 0.05, 3.89 | *NA* | 0.26, 9.72 | 0.01, 8.17 | *NA* | 0.05, 9.31 | 0.07, 3.07 | *NA* | 0.04, 8.83 |
| *P* value | 0.44 | *NA* | 0.65 | 0.44 | *NA* | 0.61 | 0.45 | *NA* | 0.76 | 0.51 | *NA* | 0.73 |

Table S2. Difference in change of attitudes towards fruit and vegetable intake and smoking between overweight/obese women compared to normal weight women in the ART intervention, ART control and natural conception intervention groups after 12 and 24 weeks of Smarter Pregnancy enrollment.

^a^ Model adjusted for age, pregnancy and respective baseline attitudes.

^b^ INT: intervention.

^c^ N of ART intervention, ART control and natural conception intervention groups, respectively: N= 556, 215 and 497.

^d^ OR: odds ratios for negative attitudes in overweight/obese women compared to normal weight women.

^e^ CI: confidence interval.

^f^ N of ART intervention, ART control and natural conception intervention groups, respectively: N=355, 162 and 333.

^g^ N of ART intervention, ART control and natural conception intervention groups, respectively: N= 93, 18 and 70.

^h^ NA: not applicable (model cannot estimate outcome due absence of women with negative attitude in ART control group).
