# Supplementary material for: The Effect of an eHealth Coaching Program (Smarter Pregnancy) on Attitudes and Practices Toward Periconception Lifestyle Behaviors in Women Attempting Pregnancy: Prospective Study
Source: J Med Internet Res. 2023 Jan 31;25:e39321. doi: 10.2196/39321 (PMC9929732; doi:10.2196/39321)
Supplement: Multimedia Appendix 3 [file jmir_v25i1e39321_app3.docx]

## **Appendix 3**

|  | Crude | | | | | | Adjusted^a^ | | | | | |
| --- | --- | --- | --- | --- | --- | --- | --- | --- | --- | --- | --- | --- |
|  | Week 12 | | | Week 24 | | | Week 12 | | | Week 24 | | |
|  | ART INT^b^ | ART control | Natural INT | ART INT | ART control | Natural INT | ART INT | ART control | Natural INT | ART INT | ART control | Natural INT |
|  |  |  |  |  |  |  |  |  |  |  |  |  |
| **Vegetables**^c^ (grams) |  |  |  |  |  |  |  |  |  |  |  |  |
| Β^d^ | -11.49 | -19.71 | -12.47 | -3.52 | -21.12 | -9.79 | -2.46 | -2.72 | -5.94 | 4.77 | -6.89 | -3.59 |
| 95% CI^e^ | -25.35, 2.38 | -39.14, -0.28 | -24.90, -0.30 | -17.83, 10.79 | -40.71, -1.52 | -23.16, 3.59 | -13.97, 9.04 | -15.92, 10.48 | -16.21, 4.33 | -7.64, 17.18 | -22.11, 8.34 | -15.14, 7.96 |
| P-value | 0.10 | 0.05 | 0.049 | 0.62 | 0.04 | 0.15 | 0.67 | 0.69 | 0.26 | 0.44 | 0.37 | 0.54 |
| **Fruit**^d,e^ (pieces) |  |  |  |  |  |  |  |  |  |  |  |  |
| β | -0.28 | -0.16 | -0.30 | -0.21 | -0.19 | -0.32 | -0.07 | 0.15 | -0.12 | -0.02 | 0.07 | -0.14 |
| 95% CI | -0.56, 0.00 | -0.58, 0.27 | -0.62, 0.01 | -0.48, 0.07 | -0.57, 0.20 | -0.596, -0.036 | -0.29, 0.14 | -0.18, 0.48 | -0.39, 0.15 | -0.26, 0.23 | -0.25, 0.38 | -0.20, 2.09 |
| P-value | 0.047 | 0.47 | 0.06 | 0.14 | 0.34 | 0.03 | 0.51 | 0.37 | 0.38 | 0.88 | 0.67 | 0.10 |
| **Smoking**^f,g^ |  |  |  |  |  |  |  |  |  |  |  |  |
| OR^h^ | 0.64 | 0.63 | 1.08 | 0.79 | 0.64 | 0.88 | 0.66 | 0.53 | 1.08 | 0.92 | 0.33 | 0.84 |
| 95% CI | 0.27, 1.53 | 0.09, 4.22 | 0.39, 3.02 | 0.32, 1.96 | 0.10, 4.11 | 0.34, 2.30 | 0.26, 1.69 | 0.05, 5.27 | 0.37, 3.19 | 0.84, 1.02 | 0.03, 4.22 | 0.31, 2.25 |
| P-value | 0.31 | 0.63 | 0.88 | 0.61 | 0.64 | 0.80 | 0.39 | 0.59 | 0.88 | 0.11 | 0.39 | 0.73 |

Table S3. Difference in change of fruit and vegetable intake and smoking between overweight/obese women compared to normal weight women in the ART intervention, ART control and natural conception intervention groups after 12 and 24 weeks of Smarter Pregnancy enrollment.

^a^ Model adjusted for age, pregnancy and respective baseline practices.

^b^ INT: intervention.

^c^ N of ART intervention, ART control and natural conception intervention groups, respectively: N= 732, 301 and 614.

^d^ β: Beta coefficient of difference in change between overweight/obese women compared to normal weight women.

^e^ CI: confidence interval.

^f^ N of ART intervention, ART control and natural conception intervention groups, respectively: N= 93, 18 and 70.

^g^ Baseline smoking behavior is not included as covariate in adjusted model.

^h^ OR: odds ratio for smoking between overweight/obese women compared to normal weight women.
