# Supplementary material for: The Effect of an eHealth Coaching Program (Smarter Pregnancy) on Attitudes and Practices Toward Periconception Lifestyle Behaviors in Women Attempting Pregnancy: Prospective Study
Source: J Med Internet Res. 2023 Jan 31;25:e39321. doi: 10.2196/39321 (PMC9929732; doi:10.2196/39321)
Supplement: Multimedia Appendix 4 [file jmir_v25i1e39321_app4.docx]

## **Appendix 4**

Table S4. Difference in change of attitudes towards fruit and vegetable intake and smoking between women participating with male partner compared to women participating alone in the ART intervention, ART control and natural conception intervention groups after 12 and 24 weeks of Smarter Pregnancy enrollment.

|  | Crude | | | | | | Adjusted^a^ | | | | | |
| --- | --- | --- | --- | --- | --- | --- | --- | --- | --- | --- | --- | --- |
|  |  | Week 12 | | Week 24 | | | Week 12 | | | Week 24 | | |
|  | ART INT^b^ | ART control | Natural INT | ART INT | ART control | Natural INT | ART INT | ART control | Natural INT | ART INT | ART control | Natural INT |
|  |  |  |  |  |  |  |  |  |  |  |  |  |
| **Vegetables**^c^ |  |  |  |  |  |  |  |  |  |  |  |  |
| OR^d^ | 1.77 | 1.60 | 0.80 | 1.47 | 1.37 | 0.85 | 2.37 | 2.59 | 0.66 | 1.86 | 1.73 | 0.75 |
| 95% CI^g^ | 1.01, 3.09 | 0.83, 3.08 | 0.35, 1.84 | 0.85, 2.55 | 0.72, 2.62 | 0.39, 1.86 | 1.17, 4.84 | 0.96, 6.98 | 0.20, 2.16 | 0.94, 3.68 | 0.69, 4.31 | 0.29, 1.98 |
| P-value | 0.04 | 0.16 | 0.59 | 0.17 | 0.34 | 0.68 | 0.02 | 0.06 | 0.481 | 0.07 | 0.24 | 0.56 |
| **Fruit**^f^ |  |  |  |  |  |  |  |  |  |  |  |  |
| OR | 0.61 | 0.78 | 0.38 | 0.73 | 1.14 | 0.52 | 0.56 | 0.63 | 0.24 | 0.67 | 1.23 | 0.38 |
| 95% CI | 0.23, 1.68 | 0.35, 1.77 | 0.10, 1.45 | 0.26, 2.08 | 0.50, 2.62 | 0.18, 1.54 | 0.18, 1.76 | 0.23, 1.73 | 0.05, 1.10 | 0.20, 2.27 | 0.38, 3.36 | 0.10, 1.42 |
| P-value | 0.33 | 0.55 | 0.15 | 0.54 | 0.76 | 0.24 | 0.31 | 0.37 | 0.06 | 0.51 | 0.83 | 0.15 |
| **Smoking**^g^ |  |  |  |  |  |  |  |  |  |  |  |  |
| OR | 2.29 | *NA*^h^ | 2.97 | 2.29 | *NA* | 2.83 | 4.42 | *NA* | 12.38 | 4.06 | *NA* | 11.25 |
| 95% CI | 0.37, 14.40 | *NA* | 0.47, 18.70 | 0.37, 14.40 | *NA* | 0.45, 17.77 | 0.57, 34.61) | *NA* | 0.26, 548.81 | 0.57, 34.47 | *NA* | 0.26, 490.03 |
| P-value | 0.38 | *NA* | 0.25 | 0.38 | *NA* | 0.27 | 0.23 | *NA* | 0.20 | 0.23 | *NA* | 0.21 |

^a^ Model adjusted for age, BMI, pregnancy and respective baseline attitudes.

^b^ INT: intervention.

^c^ N of ART intervention, ART control and natural conception intervention groups, respectively: N=570, 223 and 508.

^d^ OR: odds ratios for negative attitudes in women participating with male partner compared to women participating alone.

^e^ CI: confidence interval.

^f^ N of ART intervention, ART control and natural conception intervention groups, respectively: N= 363, 169 and 339.

^g^ N of ART intervention, ART control and natural conception intervention groups, respectively: N= 96, 20 and 71.

^h^ NA: not applicable (model cannot estimate outcome due absence of women with negative attitude in ART control group).
