# Supplementary material for: The Effect of an eHealth Coaching Program (Smarter Pregnancy) on Attitudes and Practices Toward Periconception Lifestyle Behaviors in Women Attempting Pregnancy: Prospective Study
Source: J Med Internet Res. 2023 Jan 31;25:e39321. doi: 10.2196/39321 (PMC9929732; doi:10.2196/39321)
Supplement: Multimedia Appendix 5 [file jmir_v25i1e39321_app5.docx]

## **Appendix 5**

Table S5. Difference in change in fruit and vegetable intake and smoking between women participating with male partner compared to women participating alone in the ART intervention, ART control and natural conception intervention groups after 12 and 24 weeks of Smarter Pregnancy enrollment.

|  | Crude | | | | | | Adjusted^a^ | | | | | |
| --- | --- | --- | --- | --- | --- | --- | --- | --- | --- | --- | --- | --- |
|  | Week 12 | | | Week 24 | | | Week 12 | | | Week 24 | | |
|  | ART INT^b^ | ART control | Natural INT | ART INT | ART control | Natural INT | ART INT | ART control | Natural INT | ART INT | ART control | Natural INT |
|  |  |  |  |  |  |  |  |  |  |  |  |  |
| **Vegetables**^c^ (grams) |  |  |  |  |  |  |  |  |  |  |  |  |
| Β^d^ | 7.64 | 9.45 | 18.12 | 8.13 | 14.71 | 19.65 | 2.55 | -5.66 | 17.34 | 4.01 | 2.32 | 18.86 |
| 95% CI^e^ | -4.89, 20.17 | -9.74, 28.63 | 3.26, 33.00 | -4.37, 20.62 | -3.96, 33.37 | 2.55, 36.75 | -7.59, 12.69 | -18.44, 7.12 | 5.14, 29.53 | -6.53, 14.54 | -11.30, 15.94 | 3.00, 35.73 |
| P-value | 0.23 | 0.34 | 0.02 | 0.20 | 0.12 | 0.03 | 0.62 | 0.39 | 0.006 | 0.46 | 0.74 | 0.02 |
| **Fruit**^b^ (pieces) |  |  |  |  |  |  |  |  |  |  |  |  |
| β | 0.03 | 0.03 | 0.33 | -0.02 | 0.13 | 0.40 | 0.07 | 0.21 | 0.21 | 0.02 | 0.27 | 0.28 |
| 95% CI | -0.28, 0.33 | -0.40, 0.46 | -0.03, 0.69 | -0.31, 0.26 | -0.27, 0.53 | 0.07, 0.73 | -0.18, 0.33 | -0.13, 0.55 | -0.10, 0.52 | -0.23, 0.28 | -0.07, 0.60 | -0.01, 0.57 |
| P-value | 0.851 | 0.896 | 0.07 | 0.88 | 0.53 | 0.02 | 0.56 | 0.22 | 0.18 | 0.87 | 0.11 | 0.06 |
| **Smoking**^f,g^ |  |  |  |  |  |  |  |  |  |  |  |  |
| OR^h^ | 1.03 | 0.23 | 7.54 | 0.53 | 1.14 | 1.96 | 1.12 | 0.18 | 11.19 | 0.54 | 4.06 | 2.69 |
| 95% CI | 0.42, 2.53 | 0.03, 1.62 | 0.90, 63.26 | 0.21, 1.30 | 0.18, 7.28 | 0.48, 7.95 | 0.44, 2.84 | 0.01, 3.11 | 1.17, 106.78 | 0.21, 1.36 | 0.35, 56.83 | 0.49, 14.69 |
| P-value | 0.95 | 0.23 | 0.06 | 0.16 | 0.89 | 0.35 | 0.82 | 0.24 | 0.04 | 0.19 | 0.34 | 0.25 |

^a^  Model adjusted for age, BMI, pregnancy and respective baseline practices.

^b^ INT: intervention.

^c^ N of ART intervention, ART control and natural conception intervention groups, respectively: N= 749, 311 and 631.

^d^ β: Beta coefficient of difference in practice change between women participating with male partner compared to women participating alone.

^e^ CI: confidence interval.

^f^ N of ART intervention, ART control and natural conception intervention groups, respectively: N= 96, 20 and 71.

^g^ Baseline smoking behavior is not included as covariate in adjusted model.

^h^ OR: odds ratio for smoking in women participating with male partner compared to women participating alone.
